# *Anopheles* drivers of persisting malaria transmission in Guna Yala, Panamá: an operational investigation

**DOI:** 10.1186/s12936-021-03972-z

**Published:** 2021-11-24

**Authors:** Mario I. Ávila, Élodie A. Vajda, Eileen Jeffrey Gutiérrez, Daragh A. Gibson, Mariela Mosquera Renteria, Nicholas Presley, Daniel O’Reilly, Timothy A. Burton, Allison Tatarsky, Neil F. Lobo

**Affiliations:** 1 Ministerio de Salud de Panamá (MINSA), Panama City, República de Panamá; 2 Malaria Elimination Initiative (MEI), University of California, San Francisco (UCSF), USA; 3 Clinton Health Access Initiative (CHAI), Panama City, Panama; 4 Eck Institute for Global Health, University of Notre Dame (UND), Notre Dame, IN USA

**Keywords:** Bionomics, *Ny. albimanus*, Exophagic, Malaria, Bionomia, *Ny. albimanus*, Exofágico, Malaria

## Abstract

**Background:**

Though most of Panamá is free from malaria, localized foci of transmission persist, including in the Guna Yala region. Government-led entomological surveillance using an entomological surveillance planning tool (ESPT) sought to answer programmatically-relevant questions that would enhance the understanding of both local entomological drivers of transmission and gaps in protection that result in persisting malaria transmission to guide local vector control decision-making.

**Methods:**

The ESPT was used to design a sampling plan centered around the collection of minimum essential indicators to investigate the relevance of LLINs and IRS in the communities of Permé and Puerto Obaldía, Guna Yala, as well as to pinpoint any remaining spaces and times where humans are exposed to *Anopheles* bites (gaps in protection). Adult *Anopheles* were collected at three time points via human landing catches (HLCs), CDC Light Traps (LT), and pyrethrum spray catches (PSCs) during the rainy and dry seasons. Mosquitoes were identified to species via molecular methods. Insecticide susceptibility testing of the main vector species to fenitrothion was conducted.

**Results:**

In total, 7537 adult *Anopheles* were collected from both sites. Of the 493 specimens molecularly confirmed to species, two thirds (n = 340) were identified as *Nyssorhynchus albimanus*, followed by *Anopheles aquasalis.* Overall *Anopheles* human biting rates (HBRs) were higher outdoors than indoors, and were higher in Permé than in Puerto Obaldía: nightly outdoor HBR ranged from 2.71 bites per person per night (bpn) (Puerto Obaldía), to 221.00 bpn (Permé), whereas indoor nightly HBR ranged from 0.70 bpn (Puerto Obaldía) to 81.90 bpn (Permé). Generally, peak biting occurred during the early evening. The CDC LT trap yields were significantly lower than that of HLCs and this collection method was dropped after the first collection. Pyrethrum spray catches resulted in only three indoor resting *Anopheles* collected. Insecticide resistance (IR) of *Ny. albimanus* to fenitrothion was confirmed, with only 65.5% mortality at the diagnostic time.

**Conclusion:**

The early evening exophagic behaviour of *Anopheles* vectors, the absence of indoor resting behaviours, and the presence of resistance to the primary intervention insecticide demonstrate limitations of the current malaria strategy, including indoor residual spraying (IRS) and long-lasting insecticidal nets (LLINs), and point to both gaps in protection and to the drivers of persisting malaria transmission in Guna Yala. These findings highlight the need for continued and directed entomological surveillance, based on programmatic questions, that generates entomological evidence to inform an adaptive malaria elimination strategy.

## RESUMEN

**Antecedentes:**

Aunque la mayor parte de Panamá está libre de malaria, aun persisten focos de transmisión localizados, dentro de ellos la región de Guna Yala. La vigilancia entomológica liderada por el gobierno utilizando la Herramienta de Planificación de Vigilancia Entomológica (HPVE), tuvo como objetivo responder a preguntas programáticas relevantes para lograr una mejor comprensión de los factores entomológicos de transmisión locales y las brechas en la protección que, repercuten en la transmisión persistente de la malaria para guiar la toma de decisiones de control de vectores.

**Métodos:**

En las localidades de Permé y Puerto Obaldía dentro de la región de Guna Yala, Panamá, se recolectaron* Anopheles* adultos en tres intervalos de tiempo fijos utilizando Capturas de Cebo Humano (CCH), Capturas con Trampas de Luz CDC (TL-CDC) y Capturas con Aplicación de Piretroides (CAP) durante las temporadas lluviosas y secas. Los mosquitos fueron identificados a nivel de especie mediante métodos moleculares. Se realizaron pruebas de susceptibilidad al fenitrotión en la especie principal del vector.

**Resultados:**

En total, se recolectaron 7.537 adultos de* Anopheles* en ambos sitios. De los 493 especímenes con confirmación a nivel de especie, dos tercios (n = 340) fueron identificados como* Nyssorhynchus albimanus*, seguido de* Anopheles aquasalis*. En general, las tasas de picadura humanas (TPH)) de* Anopheles* fueron más altas en exteriores que en interiores, y fueron más altas en Permé que en Puerto Obaldía: la TPH nocturna en el exterior osciló entre 2,71 picaduras por persona por noche (ppn) (Puerto Obaldía) y 221,00 ppn (Permé), mientras que la TPN nocturna en el interior osciló entre 0,70 ppn (Puerto Obaldía) y 81,90 ppn (Permé). Generalmente, el punto más alto de picaduras se produjó durante las primeras horas de la noche. El rendimiento de las trampas de luz CDC fue significativamente más bajo, que el de las CCH. Por lo anterior, la TL CDC fue removida como método de captura después de la primera ronda de colectas. Las CAP dieron como resultado solo tres* Anopheles* en reposo en interiores recolectados. La resistencia de* Ny. albimanus* al fenitrotión fue confirmada, con solo 65,5% de mortalidad en el tiempo diagnóstico.

**Conclusión:**

El comportamiento exofágico y vespertino del vector* Anopheles*, la ausencia de comportamiento de reposo en interiores y la presencia de resistencia al insecticida de intervención primaria, demuestran las limitaciones de la estrategia actual contra la malaria, incluyendo el Rociado Residual Intradomiciliario (RRI) y los Mosquiteros Impregnados con Insecticida de Larga Duración (MTILD) y marcan tanto las brechas en la protección como los factores que promueven la transmisión persistente de la malaria en Guna Yala. Estos hallazgos destacan la necesidad de una vigilancia entomológica continua y dirigida, basada en preguntas programáticas, que genere evidencia entomológica para informar una estrategia adaptativa de eliminación de la malaria.

## Background

The global burden of malaria has been substantially reduced over the last 20 years. Malaria case incidence (cases per 1000 population at risk) declined from 80 in 2000 to 58 in 2015 and 57 in 2019. However, while malaria case incidence dropped by 27% from 2000 to 2015, malaria case incidence dropped by less than 2% from 2015 to 2019, signalling a stalling rate of decline. In the Americas, malaria case incidence declined by 57% between 2000 and 2019. However, the region’s recent progress has been impacted by the drastic increase in malaria in Venezuela (cases increased from 35,500 in 2000, to over 467,000 in 2019) [1].

Understanding why and where transmission is persisting, while also ensuring effective and appropriate vector control, in tandem with appropriate access to diagnosis and treatment, are critical to accelerating progress towards malaria elimination [2]. Entomological surveillance helps monitor vector species and their population dynamics over time, as well as behavioural traits that impact disease transmission and intervention effectiveness. Entomological surveillance is a key component for identifying drivers of disease and for providing actionable evidence for intervention strategies and policy. For national malaria programmes, knowledge of local vector bionomics through question-driven entomological surveillance is critical to guide the selection of appropriate malaria interventions, the appropriate targeting of these interventions, and the management of expectations of the effects of vector control on local malaria transmission [3].

Panamá sought to achieve malaria elimination by 2020 through a focused strategy of epidemiological and entomological surveillance and targeted intervention responses in transmission foci [4–6]. Panamá is considered a ‘low transmission’ country. However, localized foci of transmission persist, marked by a seasonal epidemic largely due to the malaria parasite *Plasmodium vivax*, which accounts for 90% of detected malaria cases in Panamá [7]. While in 2017, the incidence of *P. vivax* in Panamá fell below 0.25 cases per 1000 persons, this downward trend came to a halt [8], and in 2019, Panamá was among the 12 countries in Latin America to have seen an increase in malaria case incidence of over 40% compared to 2015 [1]. In 2020, it was clear that Panamá would not meet its 2020 malaria elimination goal [7], and the country readjusted its target malaria elimination year to 2025.

Today, malaria remains a major source of morbidity in Panamá’s indigenous territories, the Comarcas [9, 10]. Traditionally, the highest burden of malaria is found in the Comarca Guna Yala, an autonomous indigenous territory largely inhabited by the Guna people [9]. Although the Guna indigenous group comprises less than 3% of the total population of Panamá, they shoulder about 90% of the country’s malaria burden [11]. The Comarca Guna Yala’s isolated geographic location, existing language and cultural barriers [9], socio-economic marginalization [12, 13] and semi-autonomous political structure have been obstacles to effective malaria control [11]. Additionally, local drivers, including an ecotype that supports vector populations [14–16] and open and unprotected housing, leave communities vulnerable [17]. Parasite importation from bordering countries and internal migration also pose challenges to malaria elimination in Panamá and in the Comarca Guna Yala [18].

Entomological data across Panamá is inconsistent. Before 1956, entomological surveys specifically targeted the Canal Zone, an area that geographically represented under 5% of the country [14, 19]. But in 1970, concerned by malaria outbreaks, the Ministerio de Salud de Panamá (MINSA) conducted extensive entomological surveys throughout the country. These entomological studies led to the identification of 14 *Anopheles* species. The most commonly collected species (i.e., highest human biting rate) was *Anopheles* (*Nyssorhynchus*) *albimanus,* followed by *Anopheles* (*Anopheles*) *punctimacula,* and *Anopheles (Nyssorhynchus) aquasalis*. Together, these 3 species accounted for just over 90% of the total collections. The remaining 11 species accounted for just under 10% of the total catches, with species composition varying from west to east of Panamá [20].

In Guna Yala, the predominant malaria vector is *Ny. albimanus* [11], a major malaria vector across Mesoamerica and the Caribbean. This species is usually considered to be exophagic and zoophilic, biting primarily during the evening but also throughout the night, although its biting behaviour varies across its distribution [6]. Calzada et al. [11] investigated the epidemiological and entomological factors linked to a malaria outbreak in Guna Yala in 2012. Through mosquito surveys in three Guna communities along the coast of Guna Yala (Playón Chico, Achutupu, and Mamitupu), the authors found that *Ny. albimanus* was the most abundant and widespread species, followed by *An. punctimacula* and *Ny. aquasalis.* The authors also found *Ny. albimanus* to be infected with *P. vivax*, the country’s predominant circulating malaria parasite [11].

Entomological surveillance spearheaded by MINSA in Guna Yala is limited. Since 2015, no entomological investigations have occurred in Guna Yala, although other mosquito surveys led by research groups have recently occurred in the neighbouring Comarca of Madungandi [21, 22]. Vector control implemented by MINSA in Guna Yala is focused on routine indoor residual spraying (IRS) with fenitrothion [11] and/or clothianidin (as of 2019) [23] in targeted areas that are high risk for malaria transmission [11, 23]. In addition to IRS, fogging with deltamethrin [24], as well as larviciding with Vectolex (*Bacillus sphaericus*) and community-based environmental management, are applied in response to newly detected cases and outbreaks. As of 2019, MINSA and its implementing partners initiated a first pilot distribution campaign of long-lasting insecticidal nets (LLINs) and long-lasting insecticidal hammock nets (LLIHNs) in key areas of Guna Yala [10, 25]. While IRS and LLINs have been proven to be highly effective against endophilic and endophagic *Anopheles* mosquitoes [26, 27], contextual effectiveness relies on local vector bionomic characteristics [3].

The ESPT [28] is a decision-support tool for planning question-based entomological surveillance activities designed for the collection of minimum essential indicators to support cost effective, locally tailored, and evidence-based vector control. The ESPT enables malaria programmes to quantify gaps in protection, i.e., spaces and times where individuals are exposed to vector bites. This study reports on MINSA-led ESPT-based findings on assessing the programmatic effectiveness of current vector interventions (LLINs and IRS) in Guna Yala towards better understanding vector-related drivers of persisting malaria transmission. This is the first demonstration of a standardized, ESPT-based, MINSA-led, entomological surveillance programme in Panamá.

## Methods

### Applying the ESPT

The ESPT was piloted in Guna Yala with MINSA in 2018/2019. The ESPT-based entomological surveillance plan was based on MINSA’s priority programme question: are LLINs and IRS appropriate for targeting malaria vectors of Guna Yala? The ESPT was used to select question-based minimum essential entomological indicators, outline a sampling design grounded in available capacity, and served as a framework for data analysis and interpretation of findings [28].

### Study sites

Comarca Guna Yala is situated on the Caribbean coast of northeast Panamá. The Comarca is comprised of about 300,000 ha of continental forest and 480 km of coastline, flanked by coral reefs and mangrove forests. The Guna people cultivate coconuts and other crops in lands that were formerly rainforest and lowlands, which creates favorable habitat for *Anopheles* species. The mean annual temperature hovers between 26 and 27 °C, while the mean annual relative humidity and rainfall range between 78 and 90%, and 1600–3000 mm, respectively. The dry season spans from mid-December to April, and the wet season runs from May to mid-December [11].

**Fig. 1:**
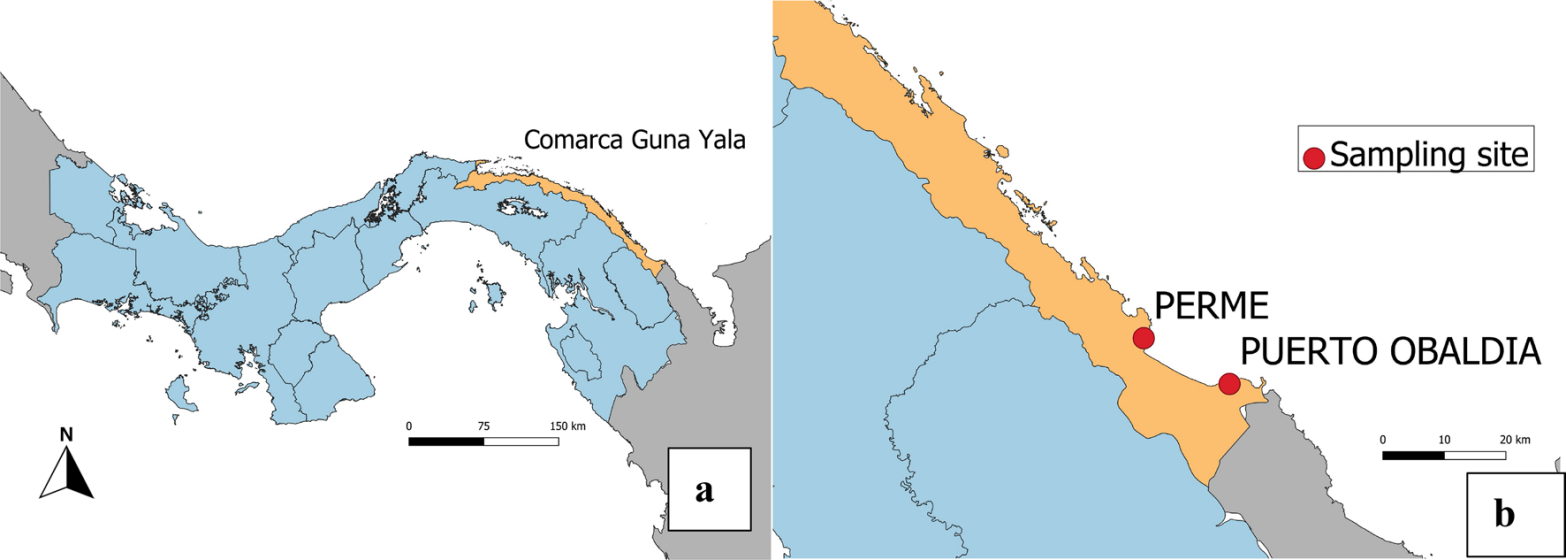
Map of Panama and the entomological sampling sites. **a** Map of Panama. **b** Map of Guna Yala with the two sampling sites

Two sentinel sites were selected for adult mosquito surveillance activities: Permé and Puerto Obaldía (Fig. 1). Permé is a Guna community with a total population of 155 inhabitants, while Puerto Obaldía is primarily an Afro-Latino community and counts 596 inhabitants. The criteria for site selection included higher incidence of reported malaria cases and representative eco-epidemiological settings in Guna Yala. In 2018 and 2019, Permé reported malaria cases year-round, with 30 cases in 2018 and 20 cases in 2019, while Puerto Obaldía reported 41 cases across 2018, and 12 cases from January to July, and in December of 2019 [29]. Both Permé and Puerto Obaldía are coastal communities with coastal lagoons, and are at the edge of the continental forest. About 19.6 km along the coastline and 16 km of sea separate Permé from Puerto Obaldía. Both communities are characterized by contrasting cultural practices and lifestyles. In Permé, homes are built of thatch rooves, bare, earthen floors, and with walls made of cane sticks secured to posts with a natural fibers [11]. In Puerto Obaldía, homes are usually constructed with cement/wooden floors and walls, and corrugated iron roofs. Up to 2018, IRS was the main vector control intervention in both communities. IRS coverage in Permé in 2018 (fenitrothion) and 2019 (clothianidin) was of 85% and 97%, respectively. In Puerto Obaldía, IRS coverage was of 97% in both 2018 and 2019, with the same insecticides as applied in Permé [30]. In 2019, LLINs were distributed in both communities, and attained a coverage of 99% in Permé and of 89% in Puerto Obaldía [31].

### Entomological sampling

Entomological sampling took place during three time points across one year to account for seasonal variation: November 2018 (moderate rainy season), March 2019 (dry season), and July/August 2019 (heavy rainy season). Adult mosquito collections occurred sequentially in Permé and Puerto Obaldía.

### Human landing catches (HLCs)

Two sentinel houses per site were sampled at each time point using HLCs [32]. Houses selected were representative of local construction. Adult mosquitoes were collected via HLCs both inside and outside houses, from 18h00 to 06h00 for seven consecutive nights in November 2018, and from 17h00 to 06h00 for five consecutive nights each in March and July/August 2019. For each HLC house, a 2-person team collected mosquitoes from 18h00 (or 17h00) to 00h00, and a second 2-person team collected from 00h00 to 06h00. One collector sampled indoors, positioned near the sleeping area of the inhabitants, and the second collector sampled outdoors, sitting about 2–5 m away from the house entrance. Each collection hour involved a 50 min collection period and a 10-min break for the collectors. To minimize collection bias, the collectors switched collection position at the end of each collection hour. Each 2-person team had one supervisor to verify quality of collections.

### Pyrethroid spray catches (PSCs)

Indoor resting mosquitoes were collected via PSCs in four houses in each site, during each of the three entomological sampling periods. For the November 2018, March and July/August 2019 PSC collections, selected houses had been last sprayed with fenitrothion in January/February 2018 (resulting in a time period greater than six months from last spray round to time of PSCs). PSC houses represented local house construction, did not overlap with HLC sentinel houses, and were at least 50 m distant from HLC houses to mitigate interactions across collection types, including the effect of the sprayed pyrethroid insecticide containing 0.125% of d-phenothrin (pyrethroid) and 0.100% of prallethrin (pyrethroid) (RAID, SC Johnson) in PSCs [32]. PSCs were only conducted in each selected house once per sampling period. Depending on when the homes were vacated by the inhabitants, PSCs started between 06h00 and 08h00, and ran for 1.5 to 2 h. At minimum, PSCs were conducted in the bedrooms.

### CDC light traps (CDC LTs)

For the CDC LT collections in November 2018, two sentinel houses were selected in each site. These were separated from the HLC houses by at least 50 m. One CDC LT was set up inside, near or in the sleeping area, and a second CDC LT was placed outside, about 2–5 m from the home entrances. Care was taken not to place the outdoor CDC LT near any other overnight light sources. One CDC LT structure in Puerto Obaldía included a border police post, where 2–6 policemen slept at a time. CDC LTs were set up at 18h00 and were stopped at 06h00 the following morning.

### Larval collections

Larval collections [32] were conducted in and around Puerto Obaldía in July 2019 for 2.5 weeks, for 4–6 h per day. All identified water bodies in and around the communities (about a 100 m periphery) were sampled to collect as many larvae as possible to ensure both sufficient *Anopheles* numbers for insecticide susceptibility testing and a diverse genetic pool.

### Insecticide susceptibility tests

Collected larvae were pooled and reared to adulthood in a temporary field insectary under natural environmental conditions. Adults were fed a 10% sugar solution. Insecticide susceptibility testing of fenitrothion was conducted with CDC Bottle Bioassays, according to the standard US CDC protocol [33]. Wheaton bottles were coated with 1 mL of fenitrothion stock solution towards a diagnostic dose of 12.5 µg/bottle. Control Wheaton bottles were coated with 1 mL of 100% ethanol. At the end of the exposure period (i.e., 2 h per test), mosquitoes were sorted by resistance phenotype (i.e., dead/knocked down or alive) at the 30-min diagnostic time, and stored in Eppendorf tubes with silica gel and cotton wool. Replicate or control number, and their corresponding status, were included on each Eppendorf tube label.

### Sample processing

Adult mosquitoes captured via HLCs, PSCs, and CDC LTs, were killed immediately following the end of the collection with RAID (SC Johnson) fumes. Dead adult mosquitoes were sorted to genus-level in the field based on morphological traits [34]. All mosquito specimens collected were counted and recorded, with only *Anopheles* samples being retained for further processing and analyses. *Anopheles* were stored in Eppendorf tubes using silica gel and cotton wool.

### Molecular identification of specimens to species-level

A randomly selected subset of samples (n = 493) from the total number of samples (n = 7537) collected through the three rounds of collections encompassing all sampling methods (November 2018, March 2019, and July/August 2019) underwent molecular analysis to confirm species identification. These samples were randomly selected over all collection periods and houses, for each of the two sites. All molecular analyses of specimens were conducted at the University of Notre Dame, USA.

Samples were sequenced at the ribosomal DNA internal transcribed spacer region 2 (ITS2) and/or cytochrome oxidase subunit 1 (*CO1*) loci towards species determination [35]. Samples were first sequenced at the ITS2 loci, and then a subset of samples with successful ITS2 sequences were also sequenced at the *CO1* loci. Final species confirmation required high sequence identity (98% or greater) to voucher sequences in multiple databases [36, 37]. *CO1* and ITS2 database comparisons for each sample were paired to determine species when either *CO1* or ITS2 alone did not produce significant results to voucher sequences [35, 36, 38–40]. Consensus sequences were manually inspected for insertions, deletions, and repeat regions to ensure these sequence differences did not inflate divergence and decrease identity scores. Consensus sequences of each sequence group were compared (BLASTn) to the NCBI nr (https://blast.ncbi.nlm.nih.gov/Blast.cgi) and BOLD [38] (https://www.boldsystems.org/) databases to identify species.

### Monthly rainfall mean

Monthly raw precipitation data were accessed online through the Empresa de Transmisión Eléctrica, S.A. (ETESA) Hydrometeoreología website [41]. Monthly rainfall means were calculated using the historical and current (2018/2019) rainfall data from the sensors stationed closest to the sampling sites. The Mulatupu sensor data and the La Miel sensor data were used to calculate the monthly rainfall means (November 2018, March, July, August 2019) as a proxy for monthly rainfall means for Permé and Puerto Obaldía, respectively.

### Statistical data analysis

Poisson regression models were generated to estimate the effect of various parameters: collection site (Permé, Puerto Obaldía), collector location (inside versus outside HLC houses), seasonal time point (i.e., collection period) on nightly *Anopheles* HBR. Models also included interaction between these parameters. The models were constructed such that the reference condition (intercept) is the mean of nightly *Anopheles* HBRs in July in Puerto Obaldía. Presented model coefficients have been exponentiated, and can be interpreted as the risk ratio (RR) associated with each parameter compared to the reference condition(s). The model intercept can be interpreted as the predicted HBR under the reference conditions. All coefficients are presented with bootstrapped 95% confidence intervals. Data analysis was conducted in R version 4.1.1 [42]. Data was cleaned, summarized, and plotted using the tidyverse packages ‘tidyr’, ‘dplyr’, and ‘ggplot2’ [43]. Generalized linear models were generated and analyzed with the ‘lme4’ and ‘arm’ packages [44, 45].

## Results

In Permé and Puerto Obaldía, *Anopheles* biting behaviour (i.e., HBR inside versus outside homes) was examined by measuring genus-level landing rates. Indoor resting behaviour was also investigated. Species identification was conducted molecularly for November 2018, March, and August/July 2019 samples.

### Molecular species confirmation of specimens from HLC catches

**Fig. 2:**
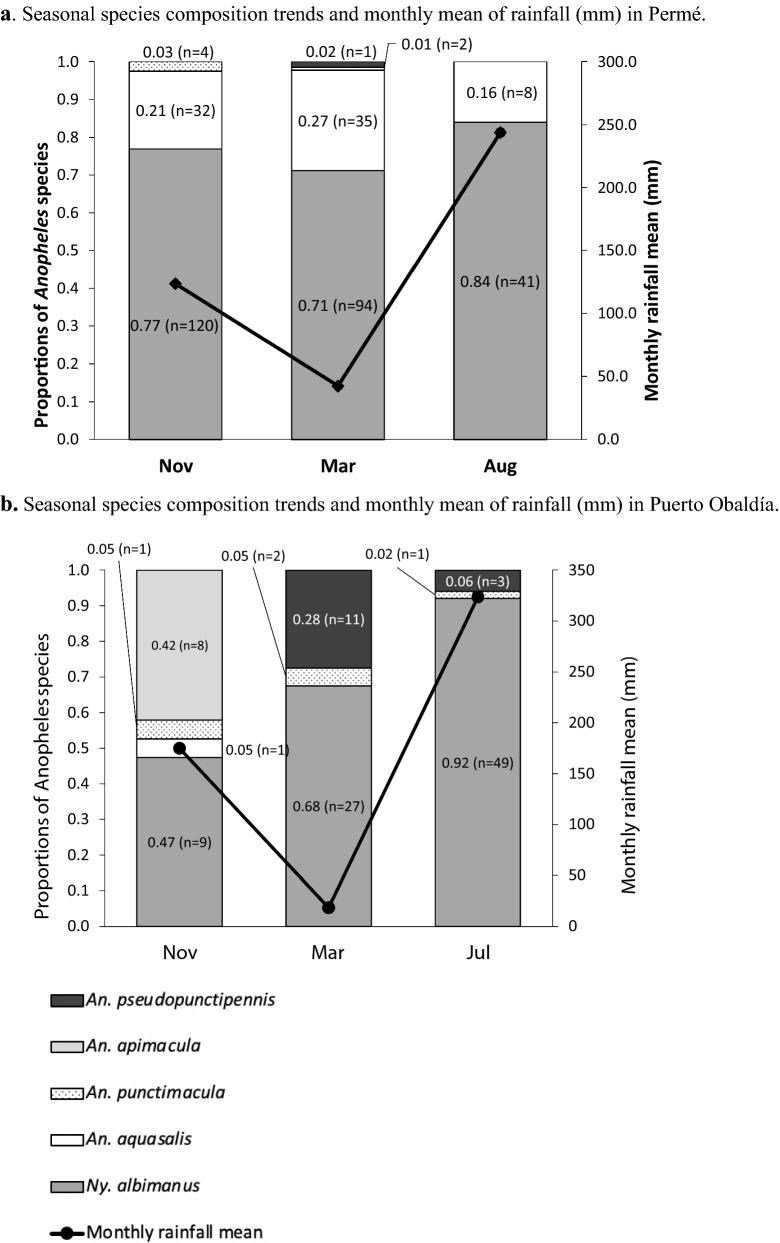
Site-specific species composition and seasonal trends in species composition in Permé (**a**) and in Puerto Obaldía (**b**). **a** Seasonal species composition trends and monthly mean of rainfall (mm) in Permé. **b** Seasonal species composition trends and monthly mean of rainfall (mm) in Puerto Obaldía

Species identification was conducted molecularly for November 2018, March and August/July 2019 samples. For the November catches, a random set of samples comprising approximately 4.5% (n = 175 specimens) of the total HLCs from both neighbouring communities ((Permé: 4.1% (n = 156 out of 3833 specimens) and (Puerto Obaldía: 38.8% (n = 19 out of 49 specimens)) were used to molecularly confirm species identification. For the March collections, a second subset of samples comprising approximately 38.3% (n = 172) of the total HLC catches (n = 449) from Permé (n = 132/383) and from Puerto Obaldía (n = 40/66) were randomly selected across all sentinel houses to molecularly confirm species identification. Finally, a third subset of samples comprising approximately 3.3% (n = 102) of the total HLC catches (n = 3,100) from Permé (n = 49 of 3029) and Puerto Obaldía (n = 53 of 71) in the July/August collection round, were also randomly selected across all sentinel houses to confirm species identification (Table 1; Fig. 2).

**Table 1:** Total number and proportion of species confirmed via molecular methods, collected by HLCs indoors and outdoors in Permé and Puerto Obaldía, Guna Yala, Panamá

Species	Anopheles species indoor n (%)	Anopheles species outdoor n (%)	Total n (%)	*Anopheles* Indoor HBR (per person per night)	*Anopheles* Outdoor HBR (per person per night)	*Anopheles* indoor:outdoor biting ratio
Permé, Nov 2018						28:109
* Ny. albimanus*	20 (65)	100 (80)	120 (77)	–	–	
* An. aquasalis*	10 (32)	22 (18)	32 (21)	–	–	
* An. punctimacula*	1 (3)	3 (2)	4 (3)	–	–	
Overall	31 (20)	125 (80)	156 (100)	**55.50**	**218.29**	
Permé, Mar 2019						
* Ny. albimanus*	19 (61)	75 (74)	94 (71)	–	–	8:31
* An. aquasalis*	12 (39)	23 (23)	35 (27)	–	–	
* An. punctimacula*	0 (0)	2 (2)	2 (2)	–	–	
* An. pseudopunctipennis*	0 (0)	1 (1)	1 (1)	–	–	
Overall	31 (23)	101 (77)	132 (100)	**7.80**	**30.50**	
Permé, Aug 2019						82:221
* Ny. albimanus*	19 (79)	22 (88)	41 (84)	–	–	
* An. aquasalis* Overall	5 (21) 24 (49)	3 (12) 25 (51)	8 (16) 49 (100)	– **81.90**	– **221.00**	
Puerto Obaldía, Nov 2018						
* Ny. albimanus*	2 (67)	7 (44)	9 (47)	–	–	1:3
* An. apimacula*	1 (33)	7 (44)	8 (42)	–	–	
* An. aquasalis*	0 (0)	1 (6)	1 (5)	–	–	
* An. punctimacula*	(0)	1 (6)	1 (5)			
Overall	3 (16)	16 (84)	19 (100)	**0.79**	**2.71**	
Puerto Obaldía, Mar 2019						
* Ny. albimanus*	3 (100)	24 (65)	27 (68)	–	–	1:6
* An. pseudopunctipennis*	0 (0)	11 (30)	11 (28)	–	–	
* An. punctimacula* Overall	0 (0) 3 (8)	2 (5) 37 (93)	2 (5) 40 (100)	– **0.70**	– **5.90**	
Puerto Obaldía, Jul 2019						
* Ny. albimanus*	11 (92)	38 (93)	49 (92)	–	–	3:5
* An. pseudopunctipennis*	1 (8)	2 (5)	3 (6)	–	–	
* An. punctimacula*	0 (0)	1 (2)	1 (2)	–	–	
Overall	12 (23)	41 (77)	53 (100)	**2.70**	**4.40**

### Species composition and biting behaviour: cross-seasonal and moderate rainy season (November 2018)

**Fig. 3:**
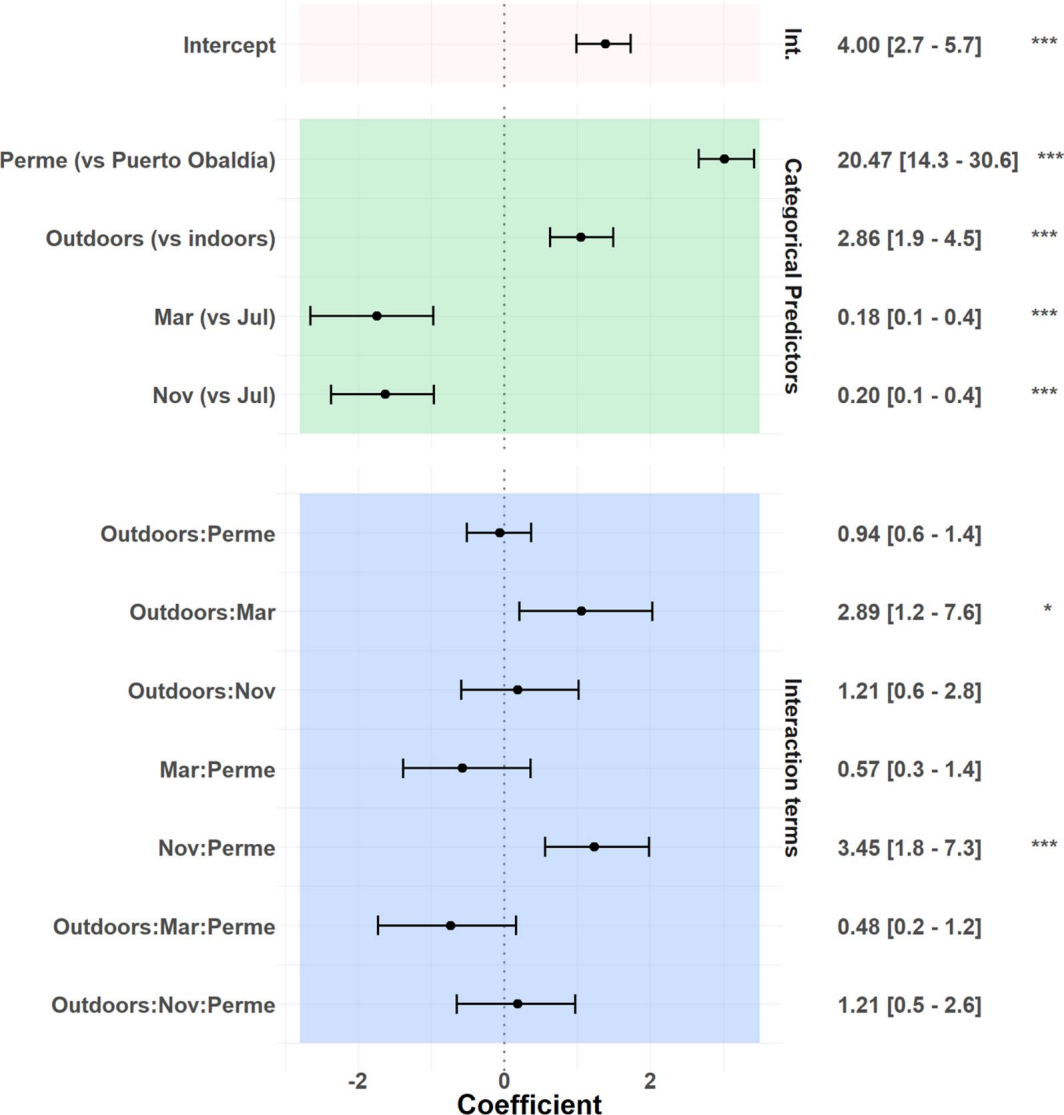
Exponentiated coefficients generated through the Poisson regression models

Across all three collection periods, nightly HBR was higher in Permé than in Puerto Obaldía (RR: 20.47 [14.3–30.6], p <  <  < 0.001). Overall outdoor nightly HBR was higher than indoor nightly HBR (RR: 2.86 ([1.9–4.5], p <  <  < 0.001) across November, March, and July/August (Fig. 3).

November occurs during the moderate rainy season; mean rainfall in November 2018 was of 123.5 mm for Permé, and 174.9 mm for Puerto Obaldía (Fig. 2). In Permé, the primary species found was *Ny. albimanus,* consisting of 77% (n = 120) of the total specimens identified to species, and *Anopheles aquasalis* comprised 21% (n = 32) of the sampled specimens. In Puerto Obaldía, the primary species was *Ny. albimanus,* consisting of 47% (n = 9), followed by *Anopheles apimacula*, comprising 42% (n = 8). Other species included *An. aquasalis* and *An. punctimacula*, each comprising 0.05% (n = 1) (Fig. 2).

**Fig. 4:**
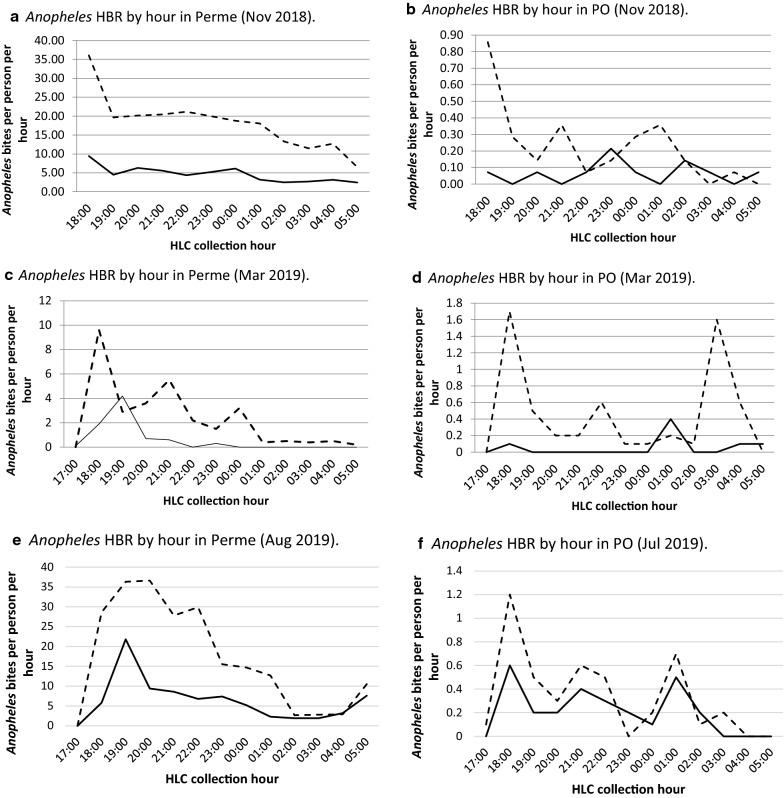
*Anopheles* HBR in Perme and PO, during the three collection periods: November 2018 (‘moderate’ rainy season), March 2019 (dry season), and July/August 2019 (‘heavy’ rainy season)

In Permé, *Anopheles* biting activity characterized by HLCs was documented throughout the night (18h00–06h00) with simultaneous outdoor and indoor biting peaks at 36.07 and 9.43 *Anopheles* bites per person per hour (bph), respectively, between 18h00 and 19h00. The primary vector identified, *Ny. albimanus*, was found host-seeking throughout the night, and is also recorded during the 18h00–19h00 biting peak. Both indoor and outdoor biting decreased at 19h00, and remained steady till 02h00, after which a noticeable decrease in biting activity was documented, followed by general decline in landing rates till 06h00 when collections ceased. Outdoor *Anopheles* landing rates were substantially higher than indoor landing rates, throughout the night (Fig. 4a). The HBR inside homes was 55.50 bpn, while the HBR outside homes was 218.29 bpn (Table 1). While mosquito activity was lower in November (moderate rainy season) than in July (heavy rainy season) in both collection sites, the difference was greater in Puerto Obaldía compared to Permé (p = 0.0006).

In Puerto Obaldía, *Anopheles* biting activity was also documented throughout the night, with the highest outdoor biting peak at 0.86 bph from 18h00 to 19h00 and two subsequent smaller peaks from 21h00 to 22h00 and 01h00 to 02h00. Indoor landing rates were substantially lower than outdoor landing rates, and were characterized by periodic landing rates of zero, the earliest being from 19h00 to 20h00 and the latest being from 04h00 to 05h00 (Fig. 4b). The HBR inside homes was 0.79 bpn, while the HBR outside homes was 2.71 bpn (Table 1). Puerto Obaldía’s primary vector species *An. albimanus*, was only noted from 18h00 to 01h00.

### Species composition and biting behaviour: dry season (March 2019)

Following the moderate rainy season, March occurs during the dry season; mean rainfall in March 2019 was of 42.3 mm for Permé, and 18.5 mm for Puerto Obaldía (Fig. 2). In Permé, the primary species found was *Ny. albimanus* constituting 71% (n = 94) of the molecularly identified specimens (n = 132). *An. aquasalis* comprised 27% (n = 35) of the molecularly identified specimens. In Puerto Obaldía, the primary species found was *Ny. albimanus* constituting 68% (n = 27), and *Anopheles pseudopunctipennis* comprised 28% (n = 11) of the molecularly identified specimens (Fig. 2).

In March, in both collection sites, outdoor nightly HBR declined but was higher than expected—relative to indoor biting—when compared to July (p = 0.021) (Fig. 3). In Permé, *Anopheles* biting activity was recorded inside and outside throughout the night, with an outdoor biting peak at 9.6 bph from 18h00 to 19h00, including *Ny. albimanus* and two subsequent, smaller peaks from 21h00 to 22h00, and from 00h00 to 01h00, after which, a steady decline in landing rates was documented until the end of the night. These recorded peak biting times all included *Ny. albimanus*. Overall, landing rates remained higher outdoors than indoors, except during the early evening peak in indoor landing rate (4.2 bph) from 19h00 to 20h00 (Fig. 4c). The HBR indoors was 7.8 mosquito bpn, versus 30.5 bpn outdoors (Table 1). Permé’s predominant vector species *Ny. albimanus’* biting activity was documented outside throughout the night starting at 18h00 till 06h00, but only until 00h00 inside.

In Puerto Obaldía, *Anopheles* biting time was also recorded throughout the night, with a first outside biting peak at 1.7 bph from 18h00 to 19h00, a second, smaller, peak between 22h00 and 23h00, and a third peak from 03h00 to 04h00. *Ny. albimanus* was noted in each of these biting peaks. *Anopheles* indoor peak biting time was at 0.4 bph from 01h00 to 02h00. As for Permé, Puerto Obaldía’s outdoor landing rates were higher than its indoor landing rates (Fig. 4d), with an indoor HBR of 0.7 bpn, and an outdoor HBR of 5.9 bpn (Table 1). Puerto Obaldía’s predominant vector, *Ny. albimanus*, was noted outdoors from 18h00 to 06h00.

### Species composition and biting behaviour: heavy rainy season (July/August 2019)

The mean rainfall in July 2019 was of 323.5 mm for Puerto Obaldía, and 243.8 mm in August 2019 for Permé, indicating the return of the heavy rains following the March dry season (Fig. 2). For both collection sites, the nightly HBRs in November and in March were significantly lower than the July nightly HBR (Fig. 3; Nov RR: 0.18 [0.1–0.4], p <  <  < 0.001; March RR: 0.20 [0.1–0.4], p <  <  < 0.001). In Permé (early August collections), the primary species found was *Ny. albimanus* consisting of 84% (n = 41). *Anopheles aquasalis* comprised 16% (n = 8) of the molecularly identified specimens. In Puerto Obaldía (end of July collections), the primary species found was *Ny. albimanus* constituting 92% (n = 49) of molecularly identified specimens. *An. pseudopunctipennis* comprised 6% (n = 3) (Fig. 2).

*Anopheles* biting activity in Permé was recorded throughout the night, with an outdoor biting peak at 36.3 bph from 19h00 to 20h00 and at 36.6 bph from 20h00 to 21h00. Permé’s indoor biting peaked at 21.8 bph from 19h00 to 20h00. From 20h00 indoors, and from 21h00 outdoors, biting activity declined steadily until the end of the night. A second, smaller outdoor biting peak was recorded from 22h00 to 23h00 (Fig. 4e). Outdoor landing rates remained higher than indoor landing rates throughout the night, whereby the HBR inside was 81.9 bpn and the HBR outside was 221.0 bpn (Table 1). Permé’s primary species collected, *Ny. albimanus*, was also present during these biting peaks.

In Puerto Obaldía, *Anopheles* biting activity was also recorded throughout the night, with two simultaneous outdoor and indoor peaks from 1.2 and 0.6 bph, respectively, from 18h00 to 19h00. Two simultaneous second outdoor and indoor biting peaks at 0.7 and 0.5 bph, respectively, occurred from 01h00 to 02h00 (Fig. 4e). Puerto Obaldía’s predominant species, *Ny. albimanus*, was also noted in these biting peaks. Throughout the night, outdoor landing rates generally remained higher than indoor landing rates (Table 1), where the HBR inside was 2.7 bpn and the HBR outside was 4.4 bpn.

### Morning indoor resting densities: November 2018, March 2019, and July/August 2019

PSCs conducted in the morning (06h00–08h00) only yielded 2 *Anopheles* from Permé in November, and 1 *Anopheles* from Puerto Obaldía in July.

### *Anopheles* densities and species composition in CDC LTs: November 2018

In November 2018, CDC LTs were tested alongside HLCs in order to determine whether or not CDC LTs could serve as a proxy for HLCs. Total nightly captures of *Anopheles* and molecular species composition in CDC LTs were compared to that of HLCs. The CDC LTs were tested alongside HLCs in November 2018, in both Permé and Puerto Obaldía. Over the course of seven collection nights via CDC LT in Permé, 106 *Anopheles* were captured both indoors and outdoors, as opposed to the total of 3,833 *Anopheles* collected via HLCs both indoors and outdoors. In Puerto Obaldía, only one *Anopheles* was collected over the course of the seven collection nights, compared with 49 collected via HLCs.

Specimens (n = 43, or 40.6% of all specimens captured from CDC LT) from Permé only were randomly selected for molecular identification. Of this subset of samples the primary species identified was *Ny. albimanus*, consisting of 55.8% (n = 24 of 43), followed by *An. aquasalis*, comprising 41.9% (n = 18 of 43), and a single *An. punctimacula*.

### Larval collections for insecticide susceptibility testing in Puerto Obaldía: species composition

Five known species of *Anopheles* from the total (n = 440) of *Anopheles* collected by larval dipping were successfully reared to the adult stage for IR testing, and confirmed through molecular species identification: *Ny. albimanus* (n = 336), *An. pseudopunctipennis* (n = 86), *An. apimacula* (n = 4), *An. punctimacula* (n = 3), and *Anopheles malefactor* (n = 2). Nine specimens were identified as ‘unknown’ *Anopheles* species.

### Insecticide susceptibility of local vectors to fenitrothion

A total of 161 female *Ny. albimanus* from the total 297 male and females collected in Puerto Obaldía were tested for susceptibility status to fenitrothion. At fenitrothion’s diagnostic time of 30 min, the obtained control mortality was 0% while the insecticide treated replicates’ mortality was 66.5%, indicating resistance to fenitrothion given that this result is below the WHO susceptibility threshold of 90% mortality.

## Discussion

Towards MINSA’s goal of understanding the vector-related drivers of persisting malaria transmission in Guna Yala, Panamá, the ESPT [28] was used to guide an operational investigation to assess the relevance of current interventions (LLINs and IRS) for targeting local malaria vectors. ESPT-based minimum essential entomological indicators were used to determine temporal species compositions and bionomic characteristics—including insecticide susceptibility, in two target communities—enabling evidence-based programmatic modifications that respond to shifting drivers of transmission.

Five *Anopheles* species were identified via molecular methods: *Ny. albimanus*, *An. aquasalis, An. apimacula, An. punctimacula*, and *An. pseudopunctipennis*. The species *Ny. albimanus* is a known major vector of *Plasmodium* in the region stretching from southern Mexico to northern South America, including in Panamá [20, 22]. The species *An. aquasalis* is also a suspected vector of *Plasmodium* in Guna Yala [20], and *An. pseudopunctipennis* and *An. punctimacula* have also been found infected with *Plasmodium* in Panamá [46–48]. The species composition remained relatively similar across both sites, with some observed differences in seasonal trends within local (community-level) species composition (Fig. 2). In both sites, *Ny. albimanus* was the most abundantly identified species from HLCs (and with CDC LTs) collections, and its proportion increased from November to July/August (Fig. 2a, b). *Anopheles pseudopunctipennis* was collected more frequently in Puerto Obaldía than in Permé (Table 1). This might be due to differences in favorable larval site availability between both sites. *Anopheles pseudopunctipennis* is a piedmont species [49] and Puerto Obaldía is directly at the foot of the mountain. In contrast, Permé is a little more distanced from the mountain and is characterized by extensive, flat wetlands (unpublished data, 2018/2019). However, the low sample size and the random sampling of molecularly identified specimens did not enable statistical analyses of these observed trends, and may have failed to document certain species present in low numbers.

At the genus-level, the interaction model depicted several subtle variations in the interactions between collector location (indoor/outdoor), collection site, and seasonal time point that were not statistically significant; these slight differences are likely due to minor weather differences across sites and to the fact that collections in Permé and Puerto Obaldía occurred sequentially, rather than simultaneously (Fig. 3). However, the regression model did confirm the statistical significance of several genus-level trends that demonstrated notable heterogeneity in *Anopheles* landing rates between the two neighbouring communities. Overall *Anopheles* HBRs remained substantially higher in Permé than in Puerto Obaldía, across all seasonal time points (Fig. 3). The reasons for this notable difference in HBR is currently unknown, although MINSA-led larval surveys in both Permé and Puerto Obaldía suggest that Permé harbours a higher number of productive larval habitats such as large brackish lagoons and wetlands (unpublished data, 2018/2019). The elevated HBR in Permé compared to Puerto Obaldía is relevant because in 2019, Permé reported more malaria cases than Puerto Obaldía: 20 cases for a total population of 155 in Permé versus 12 cases for a total population of 596 in Puerto Obaldía. Interestingly, in 2019, while malaria cases were reported in both sites during the rainy seasons, malaria cases were not any lower during the dry season (March), when nightly HBR and mean rainfall were at their lowest in both Permé and Puerto Obaldía (Fig. 2; Table 1). Indeed, three (Permé) and two (Puerto Obaldía) malaria cases were reported in both March and July (heavy rainy season). As these malaria cases are mostly accounted for by *Plasmodium vivax,* and only three by *Plasmodium falciparum* in Puerto Obaldía, a first possible explanation for this observation is that some of the *P. vivax* cases might be relapses [7, 8]. A second plausible explanation for possible dry-season malaria transmission might include human behavioural factors ensuring continued exposure to malaria vectors such that a *Plasmodium* reservoir is maintained within the communities [29]. However, the detection of *Plasmodium* in collected mosquitoes was not conducted in this investigation because in low transmission settings, sporozoite detection in even large samples of *Anopheles* may be an inefficient use of limited resources as it is highly unlikely to detect, if any, a statistically representative sample of *Plasmodium*-positive specimens [50].

Furthermore, sporozoite detection is not required from an operational perspective; MINSA does not require vector infection rates or vector incrimination data to inform vector control strategies when endemic *Anopheles* species are already known and characterized vectors. Instead, in low transmission settings such as Guna Yala, quantifying location-specific *Anopheles* landing rates is a more resource-effective and accurate way of estimating disease risk [50]. Thus, the higher landing rates observed in Permé suggest that this community has the potential to be more vulnerable to *Plasmodium* infections, which may inform intervention strategies and prioritization for this area. This observation highlights differences in prioritization of core capacity, funding and data collection requirements between programmatic and academic research. Further, possible continued malaria transmission throughout the dry season also indicates that sufficient intervention coverage during the dry season is critical in both communities to protect community members from *Plasmodium* infection.

Early evening biting behaviour was documented for *Anopheles* in both sites. In fact, daily 18h00 HLC start times in November were changed to 17h00 in March, July, and August, because November landing rates at 18h00 were not at zero, indicating earlier evening *Anopheles* biting activity. While *Anopheles* host-seeking activity was recorded throughout the night, *Anopheles* landing rates were generally higher towards the earlier evening hours (17h00–23h00), than later in the night (23h00–06h00). Hence, evidence-based changes in data collection methodologies are important to factor into surveillance towards capturing optimal and representative data. In addition, *Anopheles* landing rates were consistently higher outdoors than indoors (Fig. 3). This early evening and exophagic biting activity presents a gap in protection for community members since exophagic biting behaviour is not targeted by LLINs and IRS.

Human-vector exposure that occurs outside the exposure points targeted by LLINs and IRS is a leading cause of persistent malaria transmission in malaria endemic countries [51], and thus, additional interventions that can be used alongside LLINs and IRS and that confer additional community protection, are necessary to target outdoor biting *Anopheles.* For instance, larval source management (LSM) is a strategy that appeals to the communities of Guna Yala (MINSA, 2015), and in Columbia, nematode use to target *Ny. albimanus* resulted in a larval density decrease that was associated with a decline in malaria cases in children [52]. However, larval control is extremely laborious, costly, and logistically challenging, particularly in a densely forested region such as Guna Yala, where larval sites are plentiful, oftentimes cryptic, and dynamic. Plus, as the impact of LSM on malaria burden is not well understood [53, 54], LSM is likely not the most resource-effective intervention strategy for MINSA. On the other hand, volatile pyrethroid-based spatial repellents are a highly promising new tool that are less labor-intensive and more practical than LSM. Spatial repellents function by repelling outdoor biting vectors, and have demonstrated killing effects on the affected vectors [55–57]. Other promising novel tools include genetically modified mosquitoes [58], attractive toxic sugar baits (ATSBs) [59], and endectocides (e.g., ivermectin) [55]. The WHO Global Malaria Programme (GMP) recommends that in areas where outdoor transmission is occurring, there be a focus on evaluating the practicality, effectiveness, and affordability of novel control interventions [60]. In the specific ecological context of Guna Yala, Panamá, MINSA might consider piloting spatial repellents to address outdoor and early evening biting in the peridomestic area, as recent evidence suggests that spatial repellence have the potential to reduce malaria transmission [61].

Although bionomics data demonstrate higher outdoor and early evening biting, LLINs still do have an important protective role in Guna Yala since a proportion of biting also occurs overnight, during sleeping hours. As advised by the GMP, it is important to recognize that persisting malaria transmission despite high coverage of the core interventions (LLINs, IRS) might also signal a need to optimize the current intervention(s) already in place [60]. Susceptibility to the insecticides used, high coverage, high LLIN use, and appropriate timing of LLIN and IRS campaigns, are all key elements that MINSA must meet for these core interventions to be optimally deployed and used [51]. Further, as directed by the ESPT, to comprehensively assess the relevance of LLINs and to help identify how LLINs might be optimized, human behaviour observations (HBOs) of sleeping times and intervention use, should be integrated with the entomological data to quantify protection provided by LLINs relative to the remaining spaces and times of human-vector overlap, such as outdoor human exposure to vector biting [62].

Insecticide susceptibility testing via CDC Bottle Bioassays in Puerto Obaldía demonstrated *Ny. albimanus* resistance to fenitrothion—confirmed with only 66.5% mortality, well below the WHO threshold of 90% [63]. As of 2019, the finding of IR to fenitrothion in Puerto Obaldía enabled and validated MINSA’s switch to clothianidin (SumiShield 50WG) for IRS in Guna Yala. Clothianidin was also introduced in the two additional Guna comarcas in Panama starting in 2019, while other malarious areas continued to receive IRS using fenitrothion until MINSA ceased use of this insecticide and changed to clothianidin in 2021 for these additional regions. As MINSA transitions to sole application of clothianidin for IRS, it will be critical to monitor the current and emerging susceptibility patterns of common malaria vectors across Guna Yala and its neighbouring regions to better inform current and future IRS strategies.

However, morning indoor resting collections (PSCs) suggest that local *Anopheles* do not rest indoors in the early morning. It should be noted that in some instances, PSCs were not conducted in entire homes as some homeowners preferred that kitchen areas remain unsprayed with pyrethrum. This stipulation, along with many homes having open construction rendering the complete sealing of homes challenging (collectors used small white clothes to seal any opening identified), may have resulted in missed indoor resting *Anopheles*. However, the data yielded through early morning PSCs in Permé and Puerto Obaldía over several time points in multiple houses demonstrate that *Anopheles* are unlikely to rest indoors during the early morning hours. In addition, the last spray round of IRS with fenitrothion occurred in January/February 2018, which is outside the timeframe (4–6 months [64]) of IRS efficacy especially since the first round of PSCs began in November 2018. Thus, the absence of morning indoor resting mosquitoes suggests that long-term use of IRS in these communities may have led to behavioural changes resulting in more outdoor resting. It is recommended that during the next entomological surveillance collections, the programme conduct indoor aspirations throughout the night, in order to better quantify and assess whether *Anopheles* rest indoors at any point in the night towards determining the possible impact and appropriateness of IRS.

Finally, CDC LTs were tested alongside HLCs to evaluate whether or not CDC LTs could be used in lieu of HLCs to measure hourly and nightly HBR inside and outside homes in Permé and in Puerto Obaldía. HLCs are both labor and time intensive as well as costly, as they require the programme to mobilize key staff members and to hire community members to carry out the HLCs over the course of the collection period. Thus, a more resource-effective collection method would have the potential to help the programme maintain mosquito surveillance activities. In Permé, HLCs yielded 36.2 × *Anopheles* versus parallel CDC LTs collections (n = 3833 versus 106, respectively). Consequently, CDC LTs were dropped after the first round of collections (November) as CDC LT samples were far scarcer and were not comparable to HLCs. Data demonstrated that CDC LTs should not be used a proxy for HLCs in Guna Yala, Panamá, resulting in MINSA ceasing the use of CDC LTs for entomological surveillance activities.

## Conclusions

This ESPT-based operational investigation was conducted within the bounds set by programmatic capacity for entomological surveillance. These results, led and collected by the Panamanian Ministry of Health in a programmatic setting, support evidence-based vector control decision making. Analyses provide valuable insight into the biting and resting vector behaviours present in Guna Yala, supporting MINSA in its programme objective to better understand entomological drivers of transmission in Guna Yala. The collected ESPT-based entomological indicators were selected based on the programme question (efficacy of LLINs and IRS) and on how these interventions function (biting and resting indicators for evaluating LLIN and IRS efficacy, respectively). This framework enabled the programme to allocate their limited resources to the collection of minimum essential entomological indicators while ensuring the collection of meaningful and actionable data for their programme objectives. This work also demonstrated the relevance of validating mosquito sampling tools before scale-up, clearly showing that CDC LTs are not a valid proxy for HLCs. Indoor biting observed throughout the night suggests that LLINs are an appropriate intervention to target indoor, overnight *Anopheles* biting, although human behaviour data on intervention use and sleeping patterns would assist with clarifying and quantifying the extent of protection offered by LLINs. The lack of indoor resting mosquitoes and the presence of IR indicate that IRS may not be an optimal intervention for Guna Yala, as well as highlights the need for further evidence to confirming this conclusion.

Prevailing outdoor biting and resistance to the commonly used insecticide for IRS are critical vector-related factors that likely contribute to the persisting malaria transmission in the area, as current vector control strategies do not target exophilic and exophagic behaviours. However, to better understand how these vector bionomics findings can support the programme in making evidence-based decisions for targeted vector control interventions, these entomological data should be integrated with human behaviour data and intervention use to identify gaps in protection. This step is essential for Panamá to further understand and adapt the national strategy to reduce exposure to the malaria vectors, and thus, to continue making strides towards its malaria elimination goals.

## Data Availability

Data supporting the analysis, outcomes, and conclusions of this article are available upon request to the corresponding author.

## Abbreviations

ATSBs: Attractive toxic sugar baits
CDC LTs: CDC light traps
GMP: Global Malaria Programme
HLC: Human landing catch
IRS: Indoor residual spraying
LLINs: Long-lasting insecticidal nets
MINSA: Ministerio de Salud de Panamá
PSCs: Pyrethrum spray catches
RR: Risk ratios
